# Pinocembrin ameliorates post-infarct heart failure through activation of Nrf2/HO-1 signaling pathway

**DOI:** 10.1186/s10020-021-00363-7

**Published:** 2021-09-06

**Authors:** Xiuhuan Chen, Weiguo Wan, Yan Guo, Tianxin Ye, Yuhong Fo, Yazhou Sun, Chuan Qu, Bo Yang, Cui Zhang

**Affiliations:** 1 Department of Cardiology, Renmin Hospital of Wuhan University, 238 Jiefang Road, Wuchang, Wuhan, 430060 Hubei People’s Republic of China; 2 Cardiovascular Research Institute, Wuhan University, 238 Jiefang Road, Wuchang, Wuhan, 430060 Hubei People’s Republic of China; 3 Hubei Key Laboratory of Cardiology, Wuhan, 430060 People’s Republic of China

**Keywords:** Pinocembrin, Heart failure, Oxidative stress, Nrf2/HO-1

## Abstract

**Background:**

Oxidative stress is an important factor involved in the progress of heart failure. The current study was performed to investigate whether pinocembrin was able to ameliorate post-infarct heart failure (PIHF) and the underlying mechanisms.

**Methods:**

Rats were carried out left anterior descending artery ligation to induce myocardial infarction and subsequently raised for 6 weeks to produce chronic heart failure. Then pinocembrin was administrated every other day for 2 weeks. The effects were evaluated by echocardiography, western blot, Masson’s staining, biochemical examinations, immunohistochemistry, and fluorescence. In vitro we also cultured H9c2 cardiomyocytes and cardiac myofibroblasts to further testify the mechanisms.

**Results:**

We found that PIHF-induced deteriorations of cardiac functions were significantly ameliorated by administrating pinocembrin. In addition, the pinocembrin treatment also attenuated collagen deposition and augmented vascular endothelial growth factor receptor 2 in infarct border zone along with an attenuated apoptosis, which were related to an amelioration of oxidative stress evidenced by reduction of reactive oxygen species (ROS) in heart tissue and malondialdehyde (MDA) in serum, and increase of superoxide dismutase (SOD). This were accompanied by upregulation of nuclear factor erythroid 2-related factor 2 (Nrf2)/ heme oxygenase-1 (HO-1) pathway. In vitro experiments we found that specific Nrf2 inhibitor significantly reversed the effects resulted from pinocembrin including antioxidant, anti-apoptosis, anti-fibrosis and neovascularization, which further indicated the amelioration of PIHF by pinocembrin was in a Nrf2/HO-1 pathway-dependent manner.

**Conclusion:**

Pinocembrin ameliorated cardiac functions and remodeling resulted from PIHF by ROS scavenging and Nrf2/HO-1 pathway activation which further attenuated collagen fibers deposition and apoptosis, and facilitated angiogenesis.

**Supplementary Information:**

The online version contains supplementary material available at 10.1186/s10020-021-00363-7.

## Introduction

Heart failure (HF), characterized by a range of terminal clinical syndromes resulted from structural and/or functional impairments of heart, remains a global healthy burden, with myocardial infarction (MI) as the leading cause (Ziaeian and Fonarow 2016; Galli and Lombardi 2016; Rengo et al. 2013). Despite the great advances that have been achieved in therapies and in the quality of care in HF, there is an estimated prevalence of 37.7 million persons every year all over the world, along with 50% mortality at 5 years after diagnosis of HF (Go et al. 2014). The current dilemma might ascribe partly to that post-infarct HF (PIHF) consists of a wide variety of pathophysiological mechanisms to which present therapeutic methods are not able to completely refer.

Accumulating studies have shown that oxidative stress (OS) is predominantly involved in the pathological progression of PIHF (Dubois-Deruy et al. 2020; Ma et al. 2019; Bubb et al. 2017; Habtemariam 2019). OS, mainly presented by an excessive accumulation of free radicals/reactive oxygen species (ROS) containing hydroxyl radical, superoxide, nitric oxide, etc. (Dubois-Deruy et al. 2020; Habtemariam 2019), has been substantiated to be able to evoke oxidative modification or damage of lipid, proteins and DNA, with organelle disorders, inflammation, apoptosis in myocytes and interstitial fibrosis supervening (Dubois-Deruy et al. 2020; Bubb et al. 2017; Hermida et al. 2018). By virtue of the multiple detrimental effects of ROS, antioxidants has been taken into consideration in the PIHF therapies of which the mechanisms consist of direct ROS scavenging and/or stimulation of antioxidant defense containing glutathione, superoxide dismutase (SOD), catalase, etc. (Habtemariam 2019). However, it was regretful that application of some antioxidants, neither vitamin E (Lee et al. 2005) nor resveratrol (Made et al. 2015; Olesen et al. 2014), failed to meet the satisfaction in the therapy of cardiovascular diseases, evidenced by a failure in improving cardiac functions. Therefore, more efficient antioxidants are required in PIHF therapy.

**Fig. 1:**
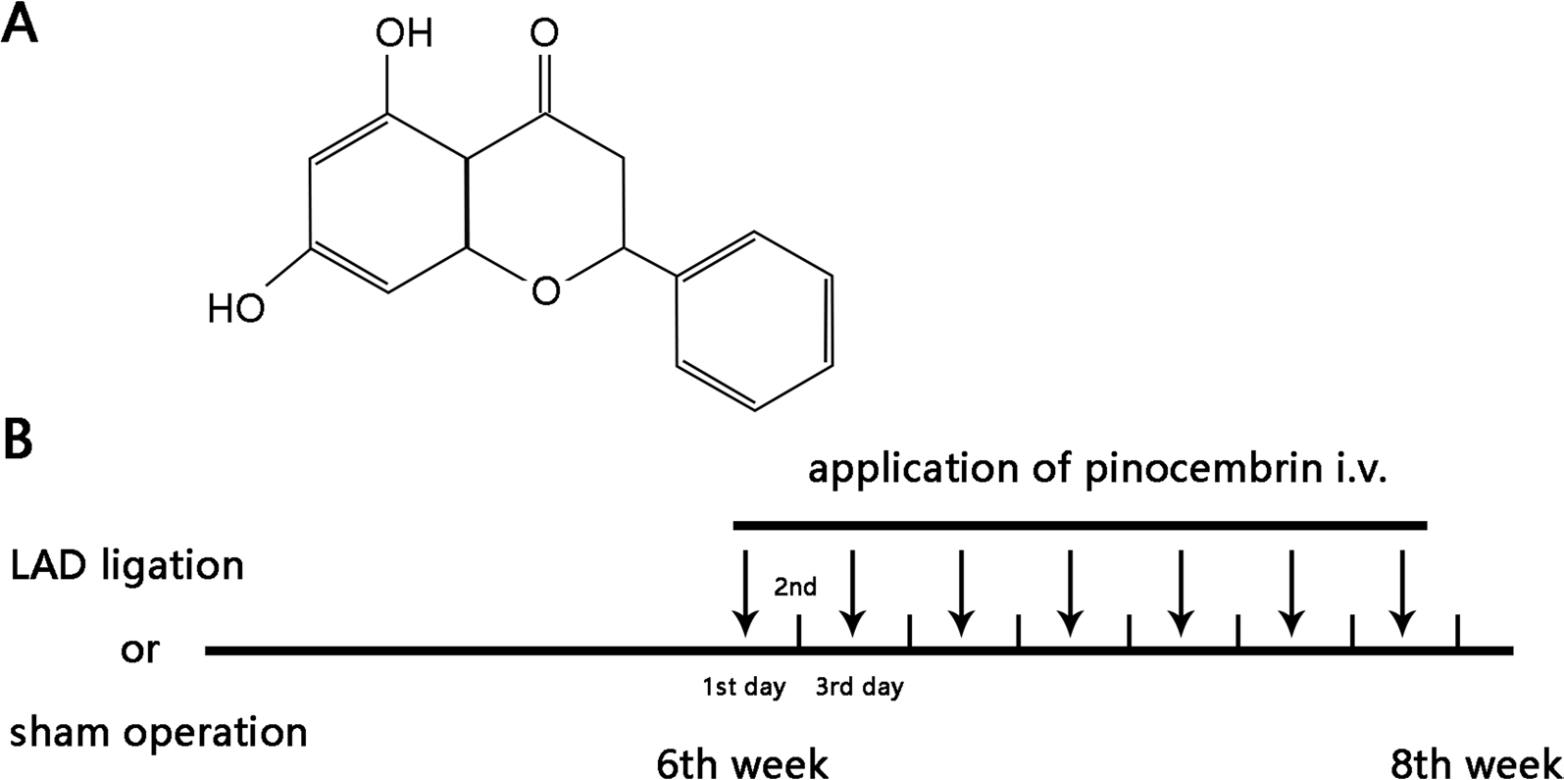
The chemical structure of pinocembrin and the animal experimental protocol. **A** The chemical structure of pinocembrin was illustrated; **B** the animal experimental protocol where an arrow meant one time of administration of pinocembrin

Pinocembrin (Pino) (the chemical structure is shown in Fig. 1A) is a natural flavonoid compound, which could be extracted from honey, propolis and several other plants (Lan et al. 2015; Rasul et al. 2013) in addition to artificial synthesis (Costa and Leitao 2011), exerting anti-inflammatory, antibacterial, anti and anticancer effects (Rasul et al. 2013; Ye et al. 2019), along with the emphasized antioxidant effect (Kim et al. 2020; Sangweni et al. 2020). As previously demonstrated, the phenolic structure of Pino responsible for direct ROS elimination as well as both phenolic and nonphenolic structures that intensify the antioxidant defense (Habtemariam 2019) account for the antioxidant effect of Pino, by which Pino has been studied and applied in a wide variety of diseases, such as neurodegenerative diseases, atherosclerosis, doxorubicin-induced cardiomyocyte toxicity (Sangweni et al. 2020; Sang et al. 2012; Liu et al. 2014). However, given that several antioxidants for myocardial infarction and HF are invalid or insufficient to convert to clinical use, whether the antioxidant effect of Pino could be fulfilled in the therapy of PIHF remains poorly understood. This research was thus performed to investigate whether administration of Pino could exert salutary effects on PIHF, holding promise to be a novel, or if not dominant but at least auxiliary treatment.

## Methods and materials

### Animal model and experiment protocol

56 male Sprague–Dawley rats, purchased from animal experiment center of China Three Gorges University, were raised in Wuhan No.3 Hospital animal administration. All experiments were officially permitted and performed in Hubei Key Laboratory of Cardiology, complied with the *Guide for the Care and Use of Laboratory Animals* published by the US National Institutes of Health (NIH Publication No. 82-23, revised in 1996).

The rats initially received left anterior descending coronary artery (LAD) ligation or sham surgery. Briefly, rats were anesthetized by 3% pentobarbital (2 mL/kg; Sigma-Aldridge, St. Louis, USA, intraperitoneal), followed by tracheal intubation and respiratory support (tidal volume: 5–10 mL, rate: 70 times/minute). Subsequent heart exposure was performed and the LAD was ligated by 7-0 monofilament nylon sutures. Occurrence of paleness of the apex cordis or ST segment elevation on II lead of electrocardiogram indicated the induction of MI. The sham operation underwent the identical procedures except the LAD ligation. Penicillin (200,000 IU) was injected intramuscularly once a day for a week after operation.

Afterwards, the rats were carefully raised for 6 weeks to produce PIHF. Rats were then randomly split into 4 groups: (i) sham + saline (sham group); (ii) sham + Pino group; (iii) LAD ligation + saline (HF group); (iv) LAD ligation + Pino (HF + Pino group). Pinocembrin (5 mg/kg, purity > 98%; Sigma-Aldridge) or same volume of saline was injected intravenously through the tail vein every other day for 2 weeks. The dosage referred to previous study (Lan et al. 2017). The protocol was illustrated in Fig. 1B.

The control positive control rats containing 3 groups: sham + saline (sham group); LAD ligation + saline (HF group); LAD ligation + enalapril (HF + ACEI group). The rats underwent corresponding operation except that the rats of HF + ACEI group were fed orally by 20 mg/kg enalapril.

### Echocardiography measurements

The cardiac functions were evaluated by transthoracic echocardiography utilizing an ultrasound imaging system (Vevo 2100, Visual Sonics Inc., Toronto, Canada), as previously reported in our group (Fo et al. 2020). Left ventricular inner dimension at end-diastolic stage (LVIDd) and end-systolic stage (LVIDs), left ventricular end-systolic and end-diastolic volume (LVESV and LVEDV) were recorded by two-dimensional M-mode echocardiograms for at least 3 consecutive cycles for each rat. Left ventricular ejection fraction (LVEF) and fractional shortening (FS), which reflected the systolic function, were subsequently calculated.

### Sample preparation

After administration of Pino and echocardiography tests, rats were anesthetized again. More than 2 mL venous blood was captured from tail vein into ethylenediamine tetraacetic acid (EDTA)-containing vacuum blood-collecting tubes with successive centrifugation at speed of 3000 r/min for 15 min and at a temperature of 4 °C, followed by isolation of serum supernatant and preservation at − 80 °C. Then the hearts were apace harvested. Part of infarct border zone of each heart was cut off and frozen in liquid nitrogen for western blot analysis, with the other heart tissue fixed in 4% paraformaldehyde for more than 24 h.

### Masson’s staining

We performed Masson’s staining to detect the collagen deposition in the infarct border zone. Paraformaldehyde-fixed ventricles were embedded by paraffin and then cut into 5-μm sections. The sections were subsequently stained with Masson trichrome where the collagen was dyed in blue, the myocardium in pink and the nucleus in black. The collagen deposition proportion was calculated by Image J software (NIH, Bethesda, MD) at × 200 magnification.

### Immunohistochemical analysis

Immunohistochemical analysis was performed to evaluate the density of vascular endothelial growth factor receptor 2 (VEGFR2) whose upregulation promoted angiogenesis that was salutary and important in attenuation of remodeling (Rengo et al. 2013). The ventricular sections were manufactured like those conducted in Masson staining, followed by deparaffinized and rehydrated. Then the sections received heat-mediated antigen retrieval and were treated with 3% H_2_O_2_ to block endogenous peroxidase, with incubation of 1 μg/mL VEGFR2 antibody (Abcam, ab9530) for 12 h at 4 ℃ fulfilled. Whereafter, the sections were incubated by horseradish peroxidase (HRP)-labeled secondary antibody (1:200, Servicebio, G1213) for 50 min at 37℃. In addition, 3,3’-diaminobenzidine was utilized to develop positive staining which was shown in tawny. Finally, the average optical density of VEGFR2 was analyzed and calculated by Image Pro Plus 6.0 software (Media Cybernetics, USA).

### Terminal-deoxynucleoitidyl transferase-mediated nick-end labeling (TUNEL) assay

Apoptosis was subsequently detected by TUNEL assay. Previously manufactured paraffin masses which embed ventricular tissue were cut into other slices for TUNEL assay, followed by TUNEL staining according to the manufacturer’s instructions of the commercial assay kit (Roche, Switzerland; 11684817910). Briefly, the ventricular sections were exposed to TUNEL reagent, where TdT and dUTP were mixed with a proportion of 1:9, for 1 h at room temperature, after incubation of proteinase K and rupture of cell membrane. Finally, the fluorescent images were observed at a wave length of 530 nm which was triggered by an emission wave length of 450 nm (× 400 magnification). The apoptotic rate of ventricular tissues in each group was analyzed and calculated by the Image J software.

### Western blot

We implement western blot to elucidate the underlying mechanisms by which Pino ameliorated PIHF. The procedures of protein sample extraction and preparation resembled those previously reported in our study (Chen et al. 2020a). The membranes were probed with antibodies against the nuclear factor erythroid 2-related factor 2 (Nrf2), heme oxygenase-1 (HO-1; 1:2000, abcam, ab189491), p53 (1:1000, cell signaling technology, 2524S), bcl-2 (1:1000, cell signaling technology, 2870P), bax (1:1000, abcam, ab32503), cleaved caspase-3 (1:1000, cell signaling technology, 9664 T), collagen I (1:1000, Affinity, AF7001), glyceraldehyde-3-phosphate dehydrogenase (GAPDH, Servicebio, GB12002), with subsequent visualization by HRP-labeled secondary antibodies (Servicebio, G1213 and G1214) and chemiluminescence detection reagents. The blot images were finally analyzed and quantified by Image J software.

### H9c2 cardiomyocytes culture and disposal in vitro

The rat heart ventricular derived H9c2 cardiomyocytes, which were purchased from the American Type Culture Collection (CRL-1446), were cultured to further verify the effects exerted by Pino in vitro. H9c2 cardiomyocytes were cultured in e Dulbecco's modified Eagle's medium (DMEM)/ F12 medium that contained 10% fetal bovine serum (Sijiqing, Hangzhou, China) under a temperature at 37 ℃ and an atmosphere of 95% air and 5% CO^2^. The cells were seeded to 6-well plates after growing to 90%, in addition, serum medium containing 25 μM isoprenaline (ISO) subsequently substituted for normal medium in 3 wells to incubate the cells for 48 h to mimic HF state since HF is also characterized by an overactivation of sympathetic tone and stimulation of β_1_-adrenergic receptor generates ROS (Velusamy et al. 2020). Then the cells were split into 6 groups: (i) control group; (ii) control medium added with 25 μM Pino for 4 h (control + Pino group); (iii) control medium co-incubated by 25 μM Pino and 5 μM ML385 for 4 h (Nrf2 inhibitor; MCE, New Jersey, USA) (control + Pino + ML385 group); (iv) ISO group; v. ISO medium with 4-h incubation by 25 μM Pino (ISO + Pino group); vi. ISO + 25 μM Pino + 5 μM ML385 group. The concentration and incubating time of drugs referred to previous study or the instructions (Jin et al. 2015; Oliveira et al. 2017).

### Neonatal rat cardiac myofibroblasts culture and proliferation assay

Neonatal rat cardiac myofibroblasts (CMFs) were cultured to investigate the effects of Pino on collagen secretion. The CMFs were separated by using neonatal rats of 3 days old, referring to previously demonstrated methods (Cui et al. 2021). The isolated and minced hearts were digested by 0.125% trypsin followed by a cocktail containing 0.25% trypsin and 0.08% collagenase for several times, with centrifugation and transplant to petri dish subsequently performed to collect the cells from the supernatant. Then the media was replaced by serum medium similar to that utilized for H9c2 cardiomyocytes culture after an adhesion of 90 min. The CMFs received identical disposals as those on H9c2 cardiomyocytes. We also implemented cell counting kit-8 (CCK-8) assay (Dojindo, Japan) to detect the proliferation of CMFs when reaching a 1.5 × 10^5^ density in each well, by exploiting microplate reader at a wave length of 450 nm, where a higher value indicated an augmented proliferation activity.

### ROS detection

The ability of Pino to scavenge ROS was measured by fluorescent probe dihydroethidium (DHE; Beyotime, Shanghai, China). DHE, which was dissolved in DMSO, was diluted to 5 μM for frozen sections or added to the serum medium to be diluted to the same concentration in accordance with the instructions. The paraformaldehyde-fixed hearts were frozen followed by being sliced up to 4-μm sections. The sections or H9c2 cardiomyocytes were co-incubated with DHE solution for 30 min at a temperature of 37 ℃, imaged afterwards by an inverted fluorescent microscope (IX70; Olympus, Tokyo, Japan). Briefly, the nucleus which were filled with DNA and RNA were dyed in distinct red due to an affinity between nucleic acid and DHE. In addition, a stronger fluorescence intensity indicated more accumulation of ROS.

### Biochemical examinations

Biochemical examinations were performed to probe the alterations of serum BNP, SOD and malondialdehyde (MDA) in serum and cellular supernatant which indicated HF phenotype and the oxidant defense, respectively. The concentration was measured by using corresponding biochemical assay kits according to manufacturer’s instructions (Jiancheng, Nanjing, China).

### Statistical analysis

The continuous variables were represented as mean ± standard error while the proportional variables were shown as percentage. Comparisons in all groups were performed by using one-way analysis of variance (ANOVA) corrected by a Tukey post hoc test except those in echocardiography data which was analyzed by two-way ANOVA corrected by the Fisher least significant difference test. A p value < 0.5 was defined as statistically significance.

## Results

### Pino ameliorated HF-induced deterioration of cardiac functions

**Fig. 2:**
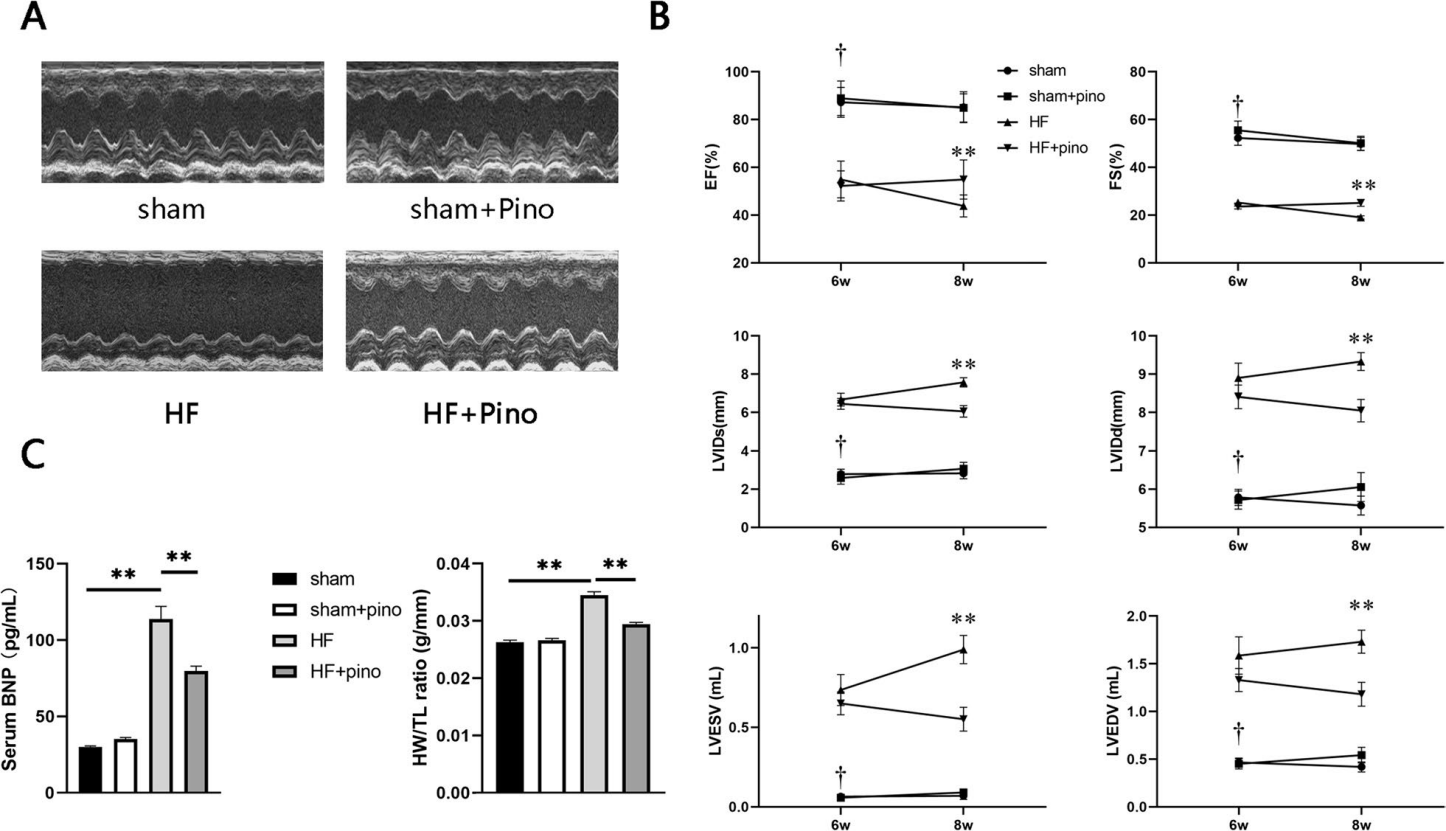
The effects exerted by chronic application of pinocembrin on cardiac functions. **A** Representative echocardiogram images in all groups; **B** the statistic analysis of parameters of cardiac functions; **C** the serum BNP concentration and heart weight/tibia length which indicated severity of heart failure and hypertrophy of heart, respectively. †: p < 0.01 when sham group compared with HF group; **: p < 0.01 when HF + Pino group compared with HF group. *BNP* brain natriuretic peptide, *HF* heart failure, *Pino* pinocembrin

We firstly implemented echocardiography to determine the HF phenotype establishment induced by LAD ligation and the effects exerted by Pino on cardiac functions. Representative two-dimension M-mode echocardiograms in all groups were illustrated in Fig. 2A. 6-week disposal-free feeding after LAD ligation produced significant HF manifestations, evidenced by reduction in LVEF and FS, and augmentation in LVIDs, LVIDd, LVESV, LVEDV at 6th and 8th week versus sham group, respectively, in addition to increased serum BNP concentration and heart weight/tibia length at 8th week that demonstrated cardiac dysfunction and hypertrophy (Fig. 2B, C), with the specific parameters were illustrated in Table 1). However, all the manifestations were partially reversed by application of Pino versus HF group at 8th week, suggesting the salutary role for Pino on cardiac functions after PIHF. Furthermore, it was noticeable that although Pino altered the baseline cardiac functions at 6th week towards a direction of improvement, the alterations did not reach significance. Therefore, an assumption was put forward that whether long-term application of Pino was able to lead to prominent ameliorations, needing further research.

**Table 1:** The cardiac parameters obtained from M-mode echocardiogram

	Sham		Sham + Pino		HF		HF + Pino	
	6w	8w	6w	8w	6w	8w	6w	8w
EF (%)	87.26 ± 2.37	85.18 ± 2.45	88.91 ± 2.73	84.87 ± 2.23	54.93 ± 2.06†	43.83 ± 1.27	52.22 ± 1.75	54.91 ± 2.27**
FS (%)	52.28 ± 3.06	49.79 ± 2.73	55.50 ± 3.84	50.13 ± 2.93	25.22 ± 1.19†	19.03 ± 0.65	23.58 ± 1.01	25.10 ± 1.37**
LVIDs (mm)	2.79 ± 0.26	2.83 ± 0.28	2.59 ± 0.32	3.07 ± 0.33	6.67 ± 0.33†	7.57 ± 0.24	6.45 ± 0.29	6.05 ± 0.30**
LVIDd (mm)	5.79 ± 0.21	5.57 ± 0.25	5.71 ± 0.24	6.06 ± 0.38	8.90 ± 0.39†	9.33 ± 0.23	8.41 ± 0.31	8.05 ± 0.29**
LVESV (ml)	0.064 ± 0.018	0.070 ± 0.024	0.057 ± 0.021	0.090 ± 0.022	0.74 ± 0.097†	0.99 ± 0.089	0.65 ± 0.071	0.55 ± 0.075**
LVEDV (ml)	0.47 ± 0.047	0.42 ± 0.053	0.45 ± 0.054	0.54 ± 0.082	1.59 ± 0.20†	1.73 ± 0.12	1.33 ± 0.12	1.18 ± 0.12**

*EF* left ventricular ejection fraction, *FS* fractional shortening, *LVIDd* left ventricular inner dimension at end-diastolic stage, *LVIDs* left ventricular inner dimension at end-systolic stage, *LVESV* left ventricular end-systolic volume, *LVEDV* left ventricular end-diastolic volume

†p < 0.01 vs. sham group at the same period; **p < 0.01 vs. HF group at the same period

### Pino attenuated the remodeling of PIHF

**Fig. 3:**
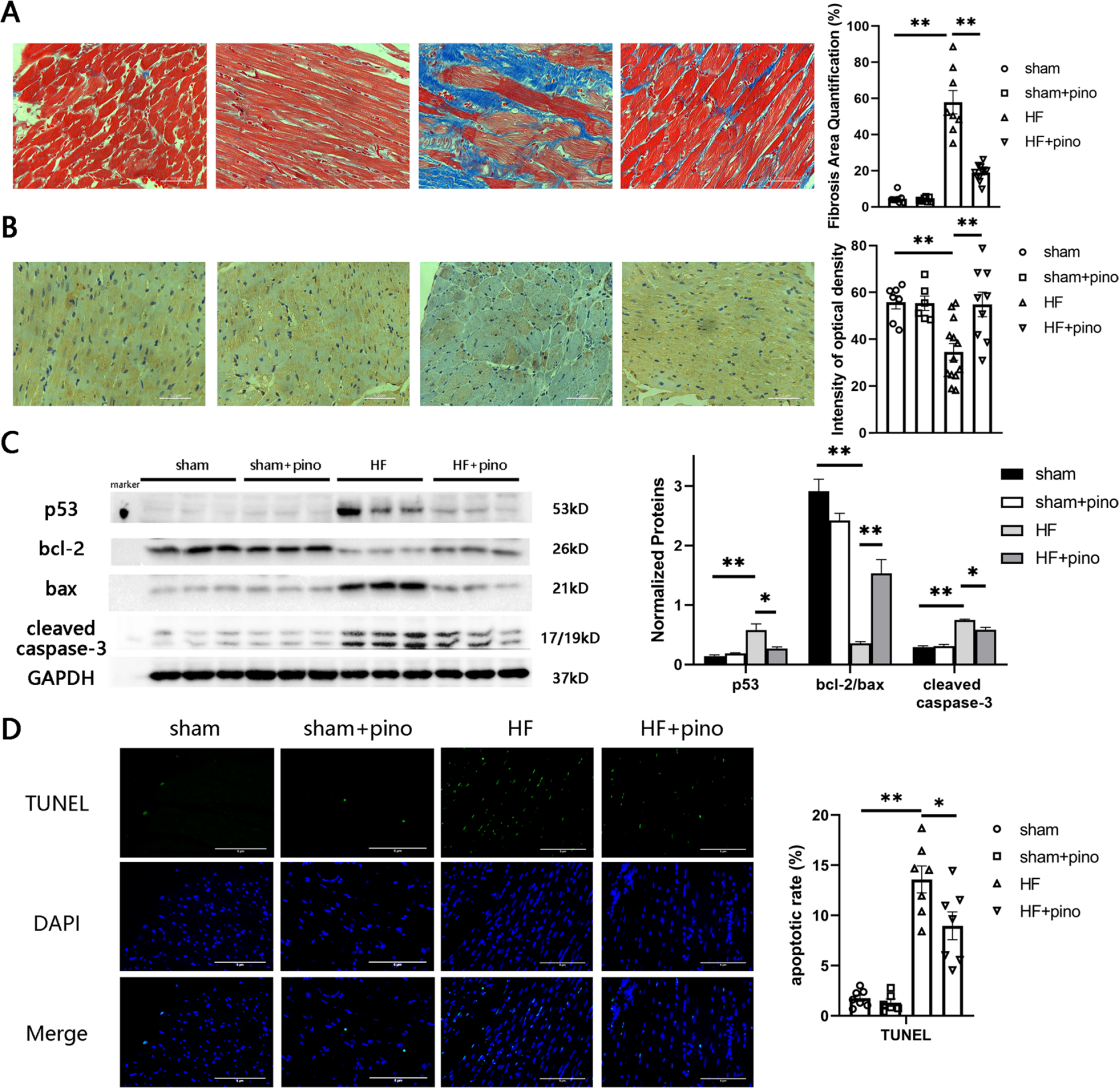
Pinocembrin alleviated post-infarct heart failure-induced ventricular remodeling. **A** pinocembrin decreased collagen deposition in infarct border zone, evidenced by the Masson staining; **B** pinocembrin reversed the heart failure-induced rarefaction of vascular endothelial growth factor receptor 2, demonstrating an amelioration of angiogenesis; **C** western blot results showed that pinocembrin improved apoptosis. N = 3 for quantified analysis of western blot. **D** Terminal-deoxynucleoitidyl Transferase-Mediated Nick-End Labeling (TUNEL) assay detected the apoptotic cardiomyocytes in infarct border zone or in the approximate region of apex cordis.*: p < 0.05; **: p < 0.01

Given that the regenerative capacity of cardiomyocytes was sparse or absent, the improvements of cardiac functions were attributed more to amelioration of the alive cardiomyocytes and tissues by Pino rather than cardiomyocytes regeneration. Thus, we further evaluated 3 key factors in PIHF remodeling: fibrosis, neovascularization and apoptosis, as previously suggested (Wang et al. 2010). Pino dramatically decreased the deposition of collagen fibers in the MI border zone by approximately 2 folds (Fig. 3A). In addition, to avoid the artificial bias in the infarct border zone judgement, we established identical model as positive control, in which Pino was replaced by enalapril, an angiotensin-converting enzyme inhibitor (ACEI) known for the reversal on HF remodeling. We observed that the fibrosis extent in HF and ACEI groups reached approximate percentage versus pre-established models. We also marked the infarct region where vast deposited collagen substituted for myocardium (Additional file 1: Figure S1). Moreover, Pino offset chronic HF-induced rarefaction of VEGFR2 distribution and expression (Fig. 3B). Previous studies have revealed that the p53 protein, which could activate diversity of pro-apoptotic genes, played a key role in anti-angiogenesis via inhibition of hypoxia inducible factor-1α (HIF-1α) (Gogiraju et al. 2019; Guo et al. 2015). We then observed an overexpression of p53 protein in HF group, which was counteracted by Pino (Fig. 3C), partly accounting for the alteration of VEGFR2. We also performed western blot experiment where administration of Pino upregulated the expression of antiapoptotic protein bcl-2 and, in contrast, downregulated the pro-apoptotic proteins including p53, bax and cleaved caspase-3, along with an increased bcl-2/bax ratio versus HF group, indicating an activation of apoptosis procedure, which was also confirmed by the TUNEL assay where Pino demonstrated a reversed effect on apoptotic rate (Fig. 3D). In a conclusion, Pino ameliorated the remodeling post MI by its well-known effects including anti-fibrosis, angiogenesis and anti-apoptosis.

### Pino alleviated OS by modulation of Nrf2/HO-1 pathways

**Fig. 4:**
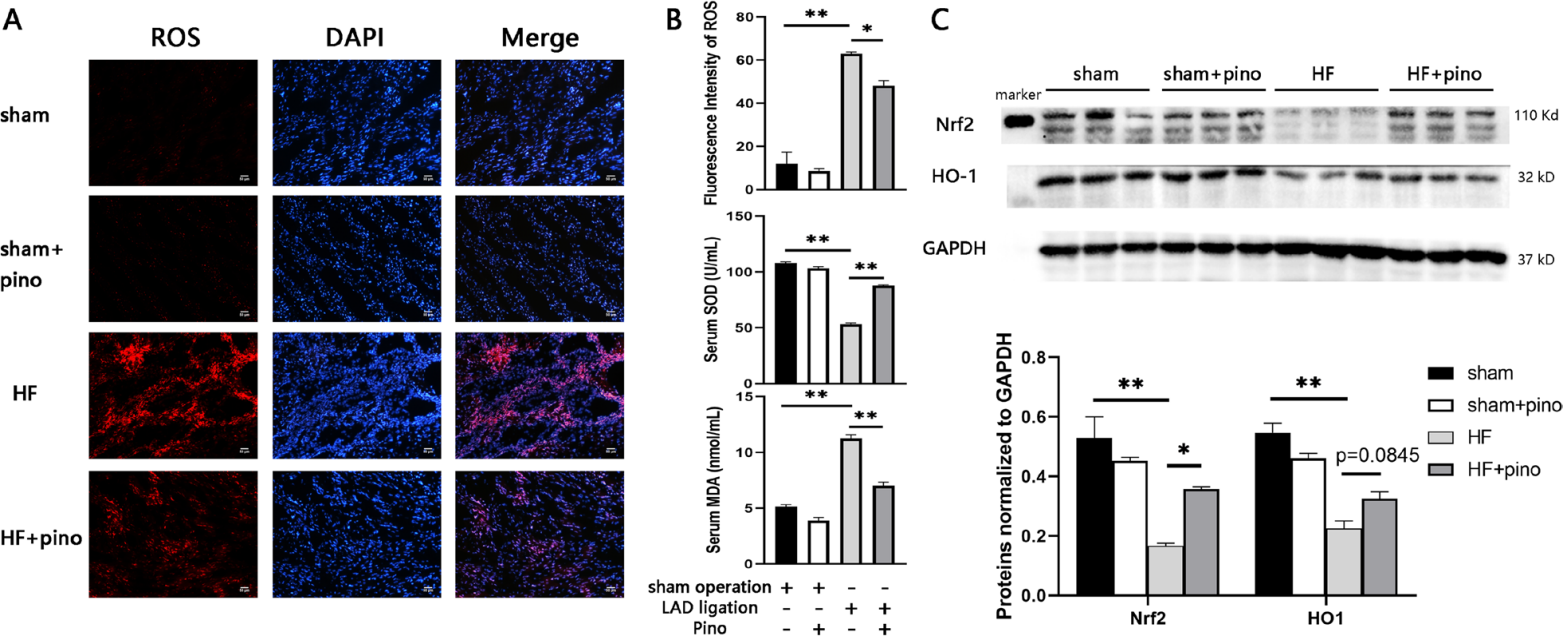
Pinocembrin ameliorated post-infarct heart failure-associated oxidative stress. **A**–**C** ROS detection, biochemical examinations and western blot results indicated that pinocembrin could scavenge ROS and facilitate antioxidant defense in a Nrf2/HO-1 signaling pathway-dependent manner. *p < 0.05; **p < 0.01. *ROS* reactive oxygen species, *Nrf2* nuclear factor erythroid 2-related factor 2, *HO-1* heme oxygenase-1

Nevertheless, the mechanisms by which Pino resulted in such alterations remained undefined. Accumulating studies have demonstrated that OS which modified a wide variety of molecules and triggered adverse remodeling signaling pathways was able to lead to apoptosis and fibrosis in HF models (Dubois-Deruy et al. 2020; Bubb et al. 2017; Wang et al. 2010; Chen et al. 2020b), we thus hypothesized that Pino ameliorated PIHF via mitigation of OS. We firstly investigated the ROS generation in heart tissues as well as SOD and MDA in serum by fluorescence and biochemical tests, respectively, to determine the antioxidant roles for Pino in HF. In expectation, Pino partially scavenged ROS generated by chronic HF, manifested by a reduction of fluorescence intensity of DHE (Fig. 4A, B). Moreover, the antioxidant enzyme, SOD was upregulated by application of Pino whereas the MDA, suggestive of lipid peroxidation, was decreased (Fig. 4B). The above data testified the antioxidant role for Pino in a rodent model of PIHF.

Albeit the ROS scavenging effect of Pino was certain, the mechanisms underlying the enhancement of antioxidant defense still necessitated elucidation. Nrf2 is a dominant component of endogenous antioxidant defense which dissociates with its inhibitor Kelch-lick ECH-associated protein 1 (Keap1) upon stresses/stimuli, and translocates to nucleus to bind to the promoter region of antioxidant response element (ARE), facilitating expression of a plethora of antioxidant genes and enzymes, among which the HO-1 was the representative enzyme and has a vital role (Ma et al. 2019; Bubb et al. 2017; Habtemariam 2019; Velusamy et al. 2020; Tian et al. 2019). HO-1, encoded by HMOX1, catalyzes heme to breakdown to 3 antioxidant molecules including biliverdin, bilirubin and carbon monoxide (CO) (Wang et al. 2010; Kishimoto et al. 2019). Previous studies have demonstrated that specific activation or overexpression of Nrf2 or HO-1 ameliorated PIHF(Ma et al. 2019; Bubb et al. 2017; Wang et al. 2010), and on the other hand, Pino could focus on Nrf2/ARE pathway to exert protective effects on depression (Wang et al. 2020), neurotoxicity (Jin et al. 2015), etc., with the interaction undefined in a PIHF model. We further investigated this interaction by western blot. PIHF contributed to reduction of expression of overall Nrf2 and HO-1 versus sham group. And Pino significantly reversed this pernicious effect by upregulation of Nrf2 in addition to a capacity to increase HO-1 expression in a strong predisposition although the difference did not reach a significance (p = 0.0845; Fig. 4C). The results indicated a role for Pino on activating Nrf2/HO-1 pathway responsible for the amelioration of cardiac functions and remodeling in PIHF.

### In vitro blockage of Nrf2 eliminated the effects of Pino in H9c2 cardiomyocytes

To further testify that the effects of Pino were Nrf2/HO-1 pathway-dependent rather than integral amelioration of HF pathological status, we cultured H9c2 cardiomyocytes as the methods reported previously, and utilized specific Nrf2 inhibitor, ML385 in vitro.

**Fig. 5:**
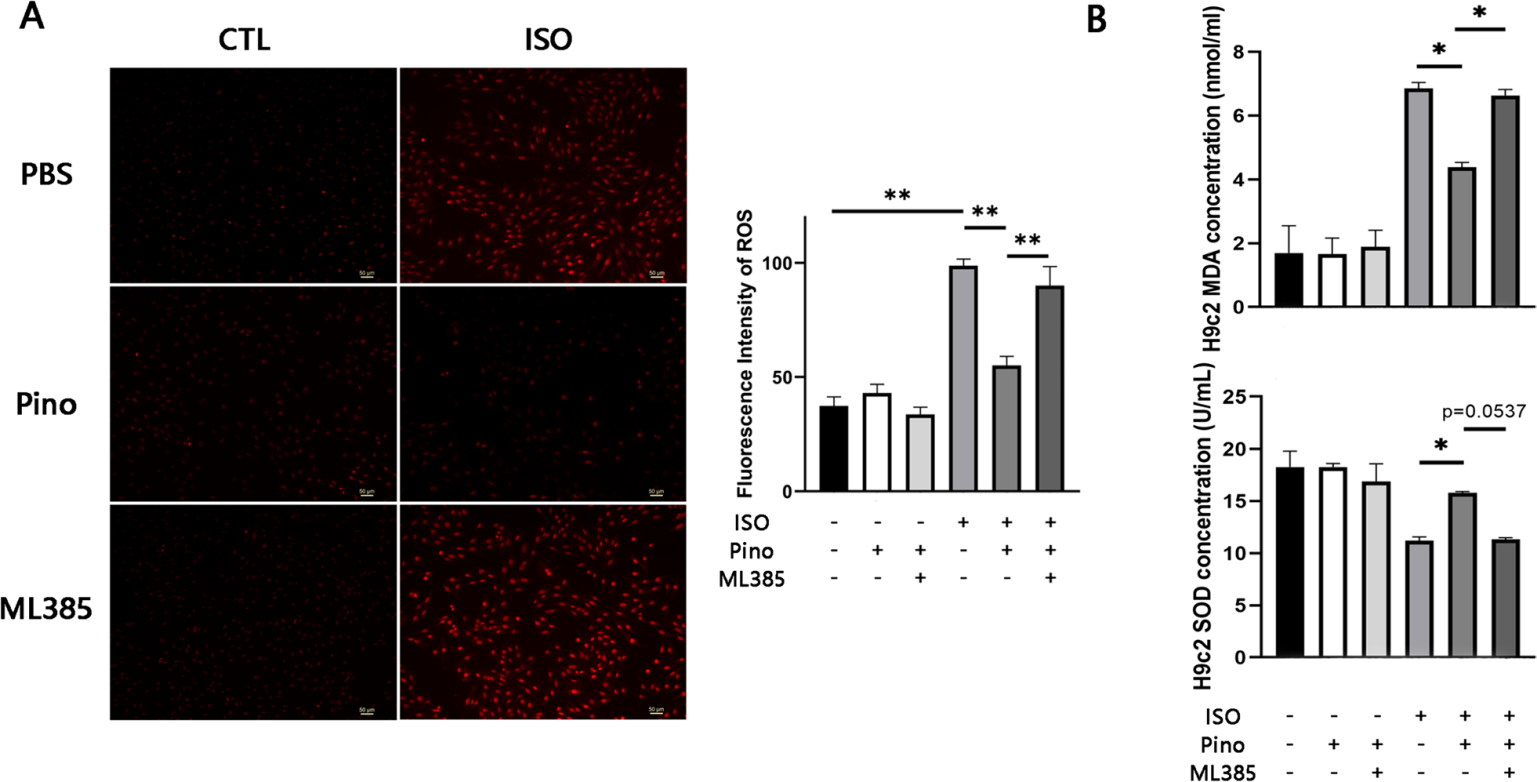
In vitro incubation of Nrf2 inhibitor 5 μM ML385 counteract the antioxidant effects of pinocembrin. **A**, **B** ROS scavenging and antioxidant defense of pinocembrin were significantly offset by the application of ML385. *p < 0.05; **p < 0.01. *ROS* reactive oxygen species, *Nrf2* nuclear factor erythroid 2-related factor 2

**Fig. 6:**
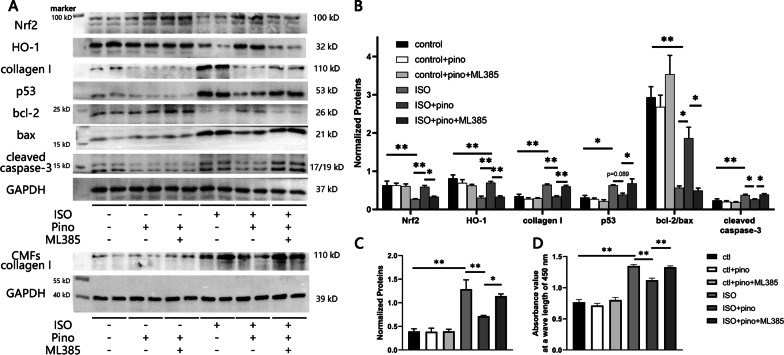
5 μM ML385 abrogated the salutary effects exerted by pinocembrin on remodeling. **A** ML385 significantly inhibited Nrf2/HO-1 signaling pathway, subsequently contributing to the exacerbation of key remodeling factors including fibrosis and apoptosis; **B** the statistic analysis for western blot. N = 4 for quantified analysis for every protein panel. **C** and **D** Quantification analysis of collagen I protein level and proliferation of CMFs. *p < 0.05; **p < 0.01. *Nrf2* nuclear factor erythroid 2-related factor 2, *HO-1* heme oxygenase-1, *CMFs* cardiac myofibroblasts

Pino attenuated ISO-induced ROS accumulation along with an increase of SOD and a decrease of MDA in the medium in a similar response pattern as those in vivo (Fig. 5). However, all effects were nearly abrogated by administration of ML385, suggesting that the antioxidant effect of Pino was predominantly presented by activation of Nrf2. Moreover, this Nrf2/HO-1 pathway-dependent effect was further determined due to the reduction of expression of Nrf2 and HO-1 in ISO + Pino + ML385 group (Fig. 6). It was also notable that the ability of ROS scavenging of Pino diminished since the efficacies of Nrf2 were mainly presented by oxidant defense. A possible explanation for this phenomenon was that an imbalance of redox homeostasis generated excessive ROS beyond the maximum scavenging ability of 25 μM Pino.

We subsequently performed H9c2 cardiomyocytes western blot to investigate the imbalance of redox homeostasis resulted from ML385 was responsible for an augmented susceptibility for remodeling. Administration of Pino showed parallel effects in anti-fibrosis, anti-apoptosis, evidenced by decrease of collagen I, p53, bax, cleaved caspase-3 as well as an increase of bcl-2 (Fig. 6A, B). Furthermore, the CCK-8 suggested an inhibition of activity of CMFs by Pino, with the reduction of collagen secretion supervening which was probed by CMFs western blot. In addition, the reversal of all these presentations by ML385 in vitro further demonstrated that Nrf2/HO-1 was the main target of Pino in amelioration of PIHF.

## Discussion

In present study, we determined that the Pino treatment was beneficial for PIHF through the antioxidant effects. Pino ameliorated cardiac functions and remodeling resulted from PIHF by ROS scavenging and Nrf2/HO-1 pathway activation which further attenuated the key factors of ventricular remodeling including collagen fibers deposition and apoptosis, and facilitated neovascularization in a p53 protein downregulation-dependent manner.

### Antioxidants AND HF

A consensus is presented that OS aggravated dysfunction and remodeling in HF (Galli and Lombardi 2016; Dubois-Deruy et al. 2020; Velusamy et al. 2020; Tian et al. 2019; Kishimoto et al. 2019), there are also a number of experiments and trials implemented for the effects of antioxidants with which more and more molecular targets and novel drugs have been discovered and studied. However, in addition to several antioxidants tested in clinical trials that predisposed to show invalidity or even increased risk for HF (Lee et al. 2005; Made et al. 2015; Olesen et al. 2014; Lonn et al. 2005), numerous molecular targets, such as the kinases, microRNAs (Tian et al. 2020; Vagnozzi et al. 2013; Pfister et al. 2014), etc., have much difficulty in transferring from basic experiments to trials, and even clinical application due to the manufacture and safety.

In contrast, Pino has following advantages promising to be an auxiliary antioxidant in therapy of HF: (i) significant antioxidant efficacy that has been testified in previous studies and our present research; (ii) Pino could be extracted from a number of natural plants, honey and propolis or be artificially synthesized which ensured the sufficiency for production and a relative cheap price; (iii) richness of Pino in honey and propolis indicated a dietary uptake for convenience. Actually, a recent phase-I clinical trial has been performed to provide Pino for healthy subjects where Pino showed well tolerance and safety (Cao et al. 2015). In addition, Pino has been approved in therapy of ischemic stroke by China Food and Drug Administration with a phase-II clinical trial being implemented (Shen et al. 2019). These achieved progresses make us look forward to further application of Pinon in more and more diseases with the involvement of PIHF.

### The roles for Pino on HF

To our knowledge, this present study was the first research to investigate the roles for Pino on a rodent model of PIHF. Although the effects of Pino were widely explored in a plethora of models, only few studies placed emphasis on the association between Pino and cardiovascular diseases including myocardial infarction, ischemia/reperfusion injury and arrhythmias (Ye et al. 2019; Zheng et al. 2020), which limited our comprehension of the protection on heart conferred by Pino. In addition, as previously demonstrated, Pino showed anti-fibrosis, -apoptosis, -inflammation and antioxidant efficacies which were also the vital targets of therapy in PIHF. Since one of the inflammation pathways, nuclear factor-kappa B/tumor necrosis factor-α (NF-κB/TNF-α) pathway has been investigated in our group (Ye et al. 2019), thus, in this study we focused on several factors that deteriorated cardiac functions and remodeling composed of apoptosis, oxidative stress, fibrosis and rarefaction of neovascularization. As expected, Pino postconditioning, which was of great significance, mitigated apoptosis and fibrosis in an antioxidant-dependent manner. We also observed VEGFR2-dependent angiogenesis that provided oxygen and nutriments for cardiomyocytes to facilitate survival, analogous to that elicited by Pino in a model of Alzheimer’s disease (Liu et al. 2014). However, a controversy remained that Pino was authenticated to suppress VEGFR2 binding, capillaries migration and sprouting (Cuevas et al. 2015; Tian et al. 2014) by which Pino could be utilized to hinder proliferation of cancer as a latent novel drug. Therefore, neovascularization in infarct border zone was probably due to the recovery of other factors rather than the direct impact by Pino. P53 protein upregulated in PIHF could explain the alteration in part. Whether Pino affected some other factors which could modulate angiogenesis, such as protein kinase B (AKT), was also unknown. Thus, the underlying mechanisms of neovascularization conferred by Pino in PIHF needed further research to be elucidated.

### Pino predominantly modulated Nrf2/HO-1 pathway in PIHF

Nrf2/HO-1 pathway was a dominant mechanism to withstand the damages caused by OS. It has been well established that Pino could ameliorated OS by promoting overall Nrf2 and HO-1 expression and Nrf2 translocation from plasm to the nucleus, which was also the reason we choose this pathway as the candidate. However, the effect exerted by Pino on Nrf2/HO-1 pathway was due to direct activation or indirect modulation after improvement of overall status remained undefined. Therefore, in this research, we determined Nrf2 as the main target of Pino through inhibition of Nrf2 by ML385 which nearly reversed the salutary effects of Pino on fibrosis, apoptosis and angiogenesis. Nevertheless, this association performed in vitro needed further investigations where Nrf2 inhibitor or siRNA or virus transfected for genes silence was necessary to be applied in vivo. Furthermore, we also observed that the ROS scavenging was suppressed by ML385 about which we put forward an assumption that an imbalanced redox homeostasis elicited excessive ROS generation beyond the capacity of Pino at a concentration of 25 μM. To testify this assumption, a concentration gradient was proposed to be arranged to detect whether sufficient Pino was able to counteract the ROS production caused by blockade of Nrf2/HO-1 pathway. Since ROS generation generally ascribed to electronic leak from mitochondrial, activated nicotinamide adenine dinucleotide phosphate oxygenase, uncoupling of nitric oxide synthase and accumulation of advanced glycosylation products, more in-depth studies could be performed to explore the detailed mechanisms by which Pino hindered ROS generation and eliminated ROS accumulation.

### Limitations of study

Present study has several limitations. We must recognize that there was a subjective bias of the definition of infarct border zone which might intensely affect the accuracy of results. To resolve this limitation, the pathological sections were chosen by 3 persons which, however, could not completely dispense with the bias. Furthermore, few western blot images showed multiple bands attributed to the polyclonal primary antibodies that had negative impacts on the quality of figures. It was also noticeable that we utilized Nrf2 inhibitor, ML385, rather than Nrf2 siRNA or adenovirus transfection, resulting in less persuasion. Last but not least, the antioxidant mechanisms of Pino were complicated, necessitating more research to further elucidate.

## Conclusion

In conclusion, administration of Pino ameliorated myocardial infarction-induced heart failure, evidenced by improvement of cardiac functions and structural remodeling including fibrosis, apoptosis and neovascularization, partially through the attenuation of OS, in a Nrf2/HO-1 pathway-dependent manner.

## Supplementary Information

**Additional file 1: Figure S1.** Masson staining result in rats of positive control groups. (A) Representative Masson staining images demonstrated collagen deposition in infarct border zone, with the infarct zone marked by arrows (black). (B) Fibrosis quantification in all groups. **: p < 0.01.

## Data Availability

The datasets used and/or analyzed during the current study are available from the corresponding author on reasonable request.

## Abbreviations

DHE: Dihydroethidium
HF: Heart failure
HO-1: Heme oxygenase-1
ISO: Isoprenaline
MDA: Malondialdehyde
MI: Myocardial infarction
Nrf2: Nuclear factor erythroid 2-related factor 2
OS: Oxidative stress
PIHF: Post-infarct heart failure
Pino: Pinocembrin
ROS: Reactive oxygen species
SOD: Superoxide dismutase
VEGFR2: Vascular endothelial growth factor receptor 2
