# Supplementary figures and images for: Pinocembrin ameliorates post-infarct heart failure through activation of Nrf2/HO-1 signaling pathway
Source: Mol Med. 2021 Sep 6;27:100. doi: 10.1186/s10020-021-00363-7 (PMC8422663; doi:10.1186/s10020-021-00363-7)

## Slide 1
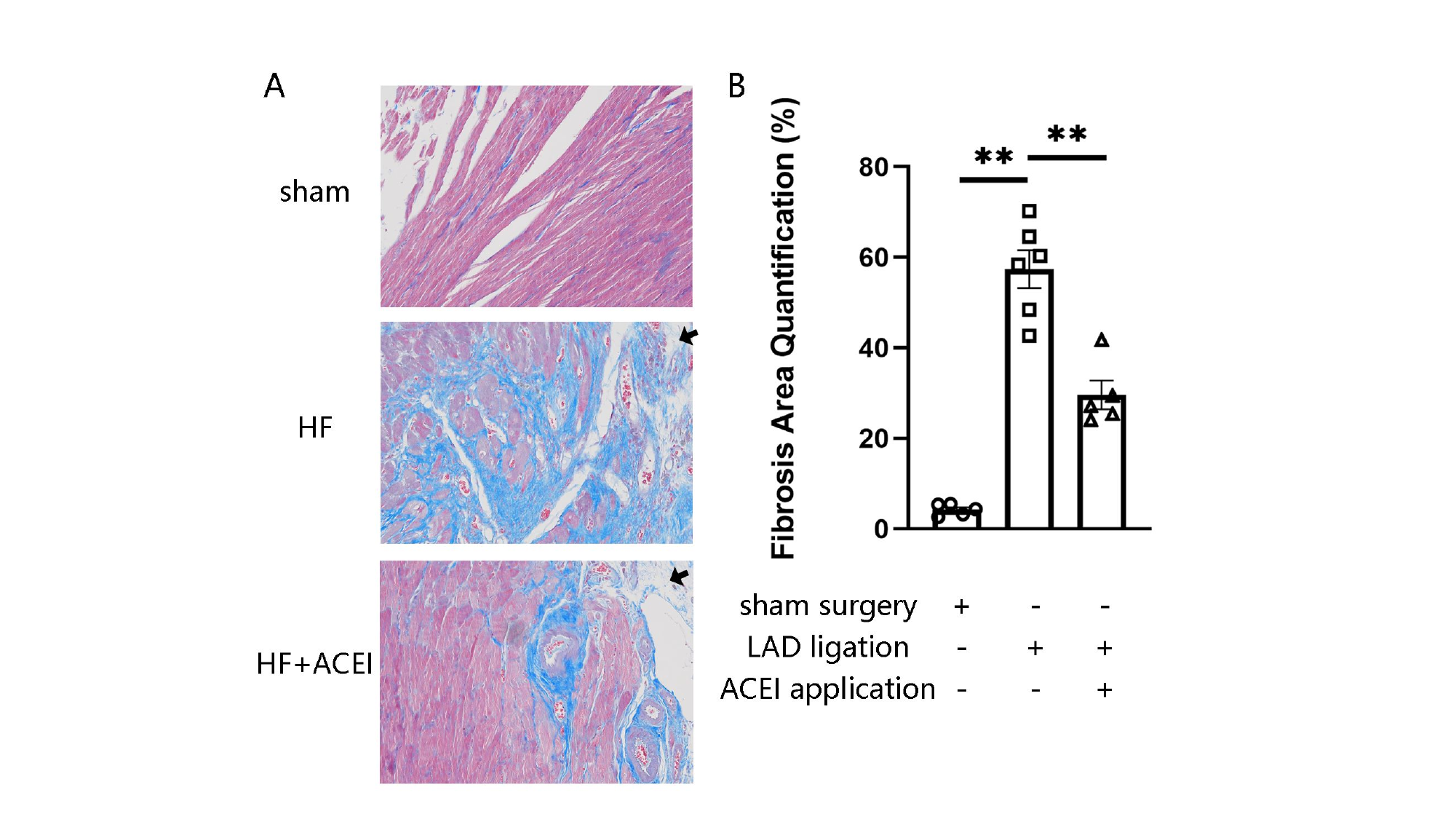

Supplement: Supplementary file 1 — Additional file 1: Figure S1. Masson staining result in rats of positive control groups. (A) Representative Masson staining images demonstrated collagen deposition in infarct border zone, with the infarct zone marked by arrows (black). (B) Fibrosis quantification in all groups. **: p < 0.01. [file 10020_2021_363_MOESM1_ESM.pptx]
